# Drug-induced renal disorders

**DOI:** 10.12861/jrip.2015.12

**Published:** 2015-09-01

**Authors:** Fatemeh Ghane Shahrbaf, Farahnak Assadi

**Affiliations:** ^1^Department of Pediatrics, Section of Nephrology, Mashhad University of Medical Sciences, Mashhad, Iran; ^2^Department of Pediatrics, Section of Nephrology, Rush University Medical Center, Chicago, Illinois, USA

**Keywords:** Acute tubular necrosis, Drugs nephrotoxicity, Interstitial nephritis, Thrombotic microangiopathy, Tubular obstruction, Hypersensitivity angeitis

## Abstract

Drug-induced nephrotoxicity are more common among infants and young children and in certain clinical situations such as underlying renal dysfunction and cardiovascular disease. Drugs can cause acute renal injury, intrarenal obstruction, interstitial nephritis, nephrotic syndrome, and acid-base and fluid electrolytes disorders. Certain drugs can cause alteration in intraglomerular hemodynamics, inflammatory changes in renal tubular cells, leading to acute kidney injury (AKI), tubulointerstitial disease and renal scarring. Drug-induced nephrotoxicity tends to occur more frequently in patients with intravascular volume depletion, diabetes, congestive heart failure, chronic kidney disease, and sepsis. Therefore, early detection of drugs adverse effects is important to prevent progression to end-stage renal disease. Preventive measures requires knowledge of mechanisms of drug-induced nephrotoxicity, understanding patients and drug-related risk factors coupled with therapeutic intervention by correcting risk factors, assessing baseline renal function before initiation of therapy, adjusting the drug dosage and avoiding use of nephrotoxic drug combinations

**Implication for health policy/practice/research/medical education:**

Drug-induced nephrotoxicity are more common among infants and young children and in certain clinical situations such as underlying renal dysfunction and cardiovascular disease. Drugs can cause acute renal injury, intra-renal obstruction, interstitial nephritis, nephrotic syndrome, and acid-base and fluid electrolytes disorders. Early detection of drugs adverse effects is important to prevent progression to end-stage renal disease. Preventive measures requires knowledge of mechanisms of drug-induced nephrotoxicity, understanding patients and drug-related risk factors coupled with therapeutic intervention by correcting risk factors, assessing baseline renal function before initiation of therapy, adjusting the drug dosage and avoiding use of nephrotoxic drug combinations.

## Introduction

Drug-induced nephrotoxicity is a common problem in clinical medicine and the incidence of drug-related acute kidney injury (AKI) may be as high as 60 percent (1-4). The condition can be costly and may require multiple interventions, including hospitalization (5). This article provides a summary of the most common mechanisms of drug-induced nephrotoxicity and prevention strategies.

Pathophysiologic mechanism of drug-induced nephrotoxicity is complex and often mediated through alteration of intraglomerular hemodynamics, impaired tubular secretion, inflammation, uric acid deposition, rhabdomyolysis, and thrombotic microangiopathy (6-8). Patients with underlying renal insufficiency, defined as glomerular filtration rate (GFR) less than 60 mL/minute/1.73 m^2^, heart failure, sepsis, and intravascular depletion are particularly vulnerable to developing nephrotoxicity (Table 1).

Aminoglycoside antibiotics, non-steroidal anti-inflammatory drugs (NSAIDs), contrast agents, and angiotensin converting enzyme inhibitors (ACEIs) are the most common cause of AKI in hospitalized patients (2). The risk of contrast-induced nephropathy is highest in diabetics and chronic kidney disease diabetes (9).

“Drugs can cause nephrotoxicity by altering intraglomeu- lar hemodynamics and decreasing GFR (ACEI, angiotensin-converting enzyme blockers [ARBs], NSAID, cyclo- sporine, and tacrolimus) (10-15).”

“Certain drugs such as ampicillin, ciprofloxacin, sulfon- amides, acyclovir, ganciclovir, methotrexate and triam- terene are associated with crystal nephropathy (16,17). Crystal nephropathy may also results from the use of che- motherapy due to uric acid and calcium phosphate crystal deposition (16,17).”

“Statins and alcohol may induce rhabdomyolysis because of a toxic effect on myocyte function, or (18-20). Drugs most often associated with thrombotic microangiopathy include antiplatelet agents (e.g., cyclosporine, mitomycin- C, and quinine (21,22).”

Drugs associated with tubular cell toxicity and acute in- terstitial nephropathy include aminoglycosides, ampho- tericin B, cisplatin, beta lactams, quinolones, rifampin, sulfonamides, vancomycin, acyclovir, and contrast agents (4,10,11). These agents induce renal tubular cell injury by impairing mitochondrial function and interfering with tubular transport and increasing oxidative stress and free radicals (6,10). Chronic use of acetaminophen, aspirin, di- uretics and lithium is associated with chronic interstitial nephritis leading to fibrosis and renal scarring (11,20-23).

## Patient-related risk factors

Drug-induced renal disorders are more common in certain patients and in specific clinical situations. Infants and young children with extracellular volume depletion, sepsis, renal impairment, cardiovascular disease, diabetes, or prior exposure to radio contrast agents are at risk of developing drug nephrotoxicity.

## Prevention strategies

Preventive strategies should target the safety of prescribing drug, monitoring their potential nephrotoxicity, correcting risk factors for nephrotoxicity.

Before initiation the drug therapy, ensure adequate hydration and avoid the use of nephrotoxic drugs whenever possible (23-25). Correct intravascular depletion to maintain renal perfusion before initiation of nephrotoxic agents (24,26). Administer drug orally and use the lowest effective dose and shortest duration of therapy whenever possible (27,28). Maintain drug levels within the recommended therapeutic range. Use less toxic analgesics with the lowest prostaglandins activity such as acetaminophen in patients with chronic pain and limit the duration of therapy. Discontinue or reduce the dose of nephrotoxic drug with the first sign of toxicity. Monitor renal function and serum drug concentrations during drug therapy.

Use the lowest dose of low osmolar contrast agent in patients with pre-existing renal insufficiency, heart failure, and diabetes. Ensure adequate hydration with normal saline or sodium bicarbonate infusion. Consider acetazolamide and monitor GFR 24-48 hours post exposure (26).

**Table 1:** The most commonly used nephrotoxic drugs

**Medication**	**Drug category**	**Renal toxicity**
Acetaminophen	Non-narcotic analgesic	Chronic interstitial nephritis, acute tubular necrosis
Acetazolamide	Carbonic-anhydrase inhibitor	Proximal renal tubular acidosis
Acyclovir	Antiviral	Acute interstitial nephritis, crystal nephropathy
Allopurinol	Hypouricemic agent	Acute interstitial nephritis
Aspirin	Non-narcotic analgesic	Chronic interstitial nephritis
Amitriptyline	Antidepressant	Rhabdomyolysis
Aminoglycosides	Antimicrobial	Acute tubular necrosis
Amphotericin B	Antifungal	Acute tubular necrosis, distal renal tubular acidosis
Angiotensin-converting enzyme inhibitors (ACEI)	Antihypertensive	Acute kidney injury
Angiotensin receptor blockers (ARB)	Antihypertensive	Acute kidney injury
Benzodiazepines	Sedative-Hypotonic	Rhabdomyolysis
Beta lactams	Antimicrobial	Acute interstitial nephritis
Carbenicillin	Antimicrobial	Metabolic alkalosis
Cephalosporin	Antimicrobial	Acute tubular necrosis
Cholpropamide	Sulfonylureas	Hyponatremia, syndrome inappropriate ADH secretion
Cimetidine	Gastrointestinal	Acute interstitial nephritis
Cisplatin	Antineoplastic	Chronic interstitial nephritis
Clopidogrel	Antiplatelet	Thrombotic miroangiopathy
Cocaine	Narcotic analgesic	Rhabdomyolysis
Contrast agents	Contrast medium	Acute tubular necrosis
Cortisone	Corticosteroid	Metabolic alkalosis, hypertension
Cyclophosphamide	Antineoplastic	Hemorrhagic cystitis
Cyclosporine	Immunosuppressive	Acute tubular necrosis, chronic interstitial nephritis, thrombotic microangiopathy
D-penicillamine	Antirheumatic	Nephrotic syndrome
Diphenhydramine	Antihistamine	Rhabdomyolysis
Furosemide	Loop diuretic	Acute interstitial nephritis
Ganciclovir	Antiviral	Crystal nephropathy
Gold Na thiomalate	Aniarthritic	Glomerulonephritis, nephrotic syndrome
Haloperidol	Antipsychotic	Rhabdomyolysis
Indinavir	Antiviral	Acute interstitial nephritis, crystal nephropathy
Interferon-alfa	Antineoplastic	Glomerulonephritis
Lansoprazole	Proton pump inhibitor	Acute interstitial nephritis
Lithium	Antipsychotic	Chronic interstitial nephritis, glomerulonephritis, rhabdomyolysis
Methadone	Narcotic analgesic	Rhabdomyolysis
Methamphetamine	Psychostimulant	Rhabdomyolysis
Methotrexate	Antineoplastic	Crystal nephropathy
Mitomycin-C	Antineoplastic	Thrombotic microangiopathy
Naproxen	Nonsteroidal anti-inflammatory	Acute and chronic interstitial nephritis, acute tubular necrosis, glomerulonephritis
Omeprazole	Proton pump inhibitor	Acute interstitial nephritis
Pamidronate acid	Bisphosphonate, osteoporosis prevention	Glomerulonephritis
Pantoprazole	Proton pump inhibitor	Acute interstitial nephritis
Penicillin G	penicillin	Glomerulonephritis
Pentamidine	Antimicrobial	Acute tubular necrosis
Phenformin	Hypoglycemic	Lactic acidosis
Phenacetin	Non-narcotic analgesic	Chronic interstitial nephritis
Phenytoin	Anticonvulsant	Acute interstitial nephritis, diabetes insipidus
Probenecid	Uricosuric	Crystal nephropathy, nephrotic syndrome
Puromycin	Antimicrobial	Nephrotic syndrome
Quinine	Muscle relaxant	Thrombotic microangiopathic
Quinolones	Antimicrobial	Acute interstitial nephritis, crystal nephropathy
Rifampin	Antimicrobial	Acute interstitial nephritis
Ranitidine	Gastrointestinal	Acute interstitial nephritis
Statins	Lipid- lowering	Rhabdomyolysis
Sulfonamides	Antimicrobial	Acute interstitial nephritis, crystal nephropathy
Tacrolimus	Immunosuppressive	Acute tubular necrosis
Tetracycline	Antimicrobial	Acute tubular necrosis
azides	Diuretic	Acute interstitial nephritis
Tolbutamide	Hypoglycemic	Nephrotic syndrome
Vancomycine	Antimicrobial	Acute interstitial nephritis

The information in this table has been obtained from numerous literature sources. For additional information on specific drugs, readers should consult the primary literature.

## Estimate of renal function

As a general rule, when a new drug is prescribed, baseline renal function should be evaluated before initiating the nephrotoxic medication. Close monitoring of renal function is also essential during the course of therapy. There are several ways to estimate GFR in children. One of the easiest and more practical one is Schwartz formula using the following formula (27):

GFR (ml/min/1.73 m^2^) = Length (cm) × k/serum creatinine (mg/dL)

k = 0.35 (infants 1-4 weeks)

k= 0.45 (4-52 weeks)

k = 0.55 (children 1-13 years)

k = 0.55 (girls 14-17 years)

k = 0.70 (boys 14-18 years)

Correct intravascular depletion to maintain renal perfusion before initiation of nephrotoxic agents (24). Use analgesics with less prostaglandin activity such as aspirin and acetaminophen. Monitor renal function and serum drug concentrations during drug therapy and use the lowest effective dose and the shortest duration of therapy whenever possible (27,28).

## Authors’ contribution

All authors contributed equally to the paper.

## Conflicts of interest

The authors declared no competitive interests.

## Ethical considerations

Ethical issues (including plagiarism, data fabrication, double publication) have been completely observed by the authors.

## Funding/Support

None.
